# Methylmalonic acid levels in serum, exosomes, and urine and its association with cblC type methylmalonic acidemia-induced cognitive impairment

**DOI:** 10.3389/fneur.2022.1090958

**Published:** 2022-12-13

**Authors:** Shuqi Sun, Hong Jin, Yu Rong, Wenqi Song, Qiliang Li

**Affiliations:** ^1^Department of Clinical Laboratory, Beijing Children's Hospital, Capital Medical University, National Center for Children's Health, Beijing, China; ^2^Department of Neurology, Beijing Children's Hospital, Capital Medical University, National Center for Children's Health, Beijing, China; ^3^Department of Rehabilitation, Beijing Children's Hospital, Capital Medical University, National Center for Children's Health, Beijing, China

**Keywords:** methylmalonic acidemia, cblC, cognitive impairment, methylmalonic acid, exosomes

## Abstract

**Background:**

The cblC type methylmalonic acidemia is the most common methylmalonic acidemia (MMA) in China. The biochemical characteristics of this disease include elevated methylmalonic acid and homocysteine (HCY), increased propionylcarnitine (C3), decreased free carnitine (C0). In this study, we aimed to clarify the roles of these biomarkers in cblC-MMA induced cognitive impairment and evaluate the capacity of methylmalonic acid in different fluids or exosomes to distinguish cblC-MMA induced cognitive impairment.

**Methods:**

15 non-inherited hyperhomocysteinemia (HHcy) patients, 42 cblC-MMA patients and 57 age- and sex-matched healthy children were recruited in this study. The levels of HCY were detected by an automatic immune analyzer. The levels of acylcarnitines and methylmalonic acid were detected by tandem mass spectrometer.

**Results:**

The main findings were all biomarkers as HCY, acylcarnitines and methylmalonic acid had capacities for distinguishing patients with cblC-MMA induced cognitive impairment from healthy children. The methylmalonic acid in different fluids or exosomes had good performances for distinguishing patients with cblC-MMA induced cognitive impairment from HHcy patients. The methylmalonic acid in serum exosomes and neuronal-derived exosomes were able to distinguishing cblC-MMA patients with cognitive impairment from patients without cognitive impairment. The methylmalonic acid in neuronal-derived exosomes might be helpful to evaluate the severity of cblC-MMA induced cognitive impairment.

**Discussion:**

Methylmalonic acid levels in serum exosomes, especially in serum neuronal-derived exosomes, serve as potential biomarkers for distinguishing cblC-MMA induced cognitive impairment.

## Introduction

Methylmalonic acidemia (MMA) is the most common organic acidemia in children. Normally, amino acid (e.g., Valine, Threonine, Isoleucine, Methionine), odd chain fatty acids and cholesterol *in vivo* are metabolized into methylmalonyl coenzyme A. Methylmalonyl coenzyme A converts to succinyl coenzyme A under the catalysis of methylmalonyl coenzyme A mutase and its coenzyme vitamin B12. MMA is caused by enzyme deficiency of methylmalonyl coenzyme A mutase or vitamin B12, which leads to the interruption from methylmalonyl coenzyme A to succinyl coenzyme A and the accumulation of methylmalonic acid. According to the serum homocysteine (HCY) levels, MMAs are divided into isolated MMA and combined MMA and homocystinuria (1, 2). The cblC type methylmalonic acidemia (cblC-MMA) is the most common MMA in China (3–5). The biochemical characteristics of cblC disease include elevated serum methylmalonic acid and HCY, increased propionylcarnitine (C3), decreased free carnitine (C0). Molecular diagnosis of cblC disease relies on the detection of MMACHC mutations (4, 5).

The cblC-MMA patients have a wide spectrum of clinical manifestations which include feeding difficulties, failure to thrive, hydrocephalus, neurological regression, neuropsychiatric symptoms, progressive encephalopathy, neurological deterioration, hypotonia. Most patients are troubled with abnormality of nervous system and cognitive impairments, which include cognitive difficulties, disabling movement disorder, language, and social development impairment (6–8). Methylmalonic acid and homocysteine are recognized as pathogenic substances of these patients (5, 9, 10). Methylmalonic acid, a major neurotoxin, can induce brain injury and cognitive impairment. Previous studies revealed that methylmalonic acid led to neuronal damage by inhibiting mitochondrial respiratory chain (11, 12), transmitochondrial malate shuttle (13), pyruvate carboxylase (14) and β-hydroxybutyrate (15) and inducing neuron apoptosis by mechanisms of oxidative stress injury (16, 17), neuroinflammation (18, 19) and DNA damage (20). Homocysteine is an intermediate thiol amino acid derived from methionine. Normally, methionine synthase catalyzes homocysteine to methionine within the methionine cycle. VitB12 enzyme deficiency leads to the interruption of methionine cycle and the accumulation of homocysteine (21). Some studies reported that homocysteine aggravated neuron injury through redox imbalance and neuronal autophagy over activation (22, 23). Though elevated homocysteine is related to cognitive impairment in adults, few studies reported whether the elevated homocysteine is associated with cognitive decline in cblC disease children.

In addition, the previous studies reported that the levels of propionylcarnitine (C3) increased, free carnitine (C0) decreased and the ratios of C3/C0 elevated in the cblC-MMA patients. However, it is unclear whether the levels of these acylcarnitines are associated with cognitive impairment in MMA patients.

Exosomes (a diameter of 30–100 nm vesicles) are intercellular communication microparticles secreted by numerous cell types, including neurons (24). Exosomes easily cross the blood-brain barrier and package pathological substances from the central nervous system to the blood due to their nanoscale size and structural similarity (24, 25). Some studies reported that neuronal-derived exosomes in patients with neurodegenerative diseases packaged several proteins as potential biomarkers associated with cognitive decline (26–28). However, the rare studies showed whether the levels of methylmalonic acid in the serum exosomes or neuro-exosomes were used as potential biomarkers of cognitive impairment induced by cblC-MMA.

Therefore, this study aimed to (1) clarify the roles of serum homocysteine, C3, C0, and C3/C0 in cblC-MMA induced cognitive impairment (2) evaluate the capacity of serum methylmalonic acid, urinary methylmalonic acid, serum total exosomes and neuro-exosomal methylmalonic acid to distinguish cblC-MMA induced cognitive impairment.

## Materials and methods

### The participants and samples

This study was conducted according to the principles expressed in the Declaration of Helsinki and approved by the ethic committee of the Medical Ethics Committee of Beijing Children's Hospital, Capital Medical University (approval ID: 2018-29). All the procedures were performed with the Ethical Standards of Medical Ethics Committee of Beijing Children's Hospital. Informed consents were obtained from the participants or legal guardians.

A total of 114 participants were recruited, including 15 non-inherited hyperhomocysteinemia (HHcy) patients, 42 cblC-MMA patients and 57 age- and sex-matched healthy individuals in the same period as controls. The non-inherited hyperhomocysteinemia patients involved abnormally high levels of homocysteine (>15 μmol/L) in the serum (29) and were not with cystathionine beta-synthase deficiency. The cblC-MMA patients were diagnosed by MMACHC mutations (9). Baseline and clinical characteristics were shown in Table 1. The cognitive assessment tests were conducted in participants by a trained interviewer using the Gesell developmental Scales (children ages < 6 years), Wechsler preschool and primary scale of intelligence, or Wechsler intelligence scales for children (between 6 and 18 years old) (30). Intelligence quotient (IQ) scores and development quotient (DQ) scores of patients were recorded. The standardized mean of the DQ or IQ is 100 with a standard deviation of 15 (31, 32). DQ scores or IQ scores < 70 is classified as cognitive impairment. DQ are classified as 55-69 as mild cognitive impairment, 40–54 as moderate cognitive impairment, scores < 40 as severe delay of cognition. IQ scores are classified as 52–69 as mild cognitive impairment, 36–51 as moderate cognitive impairment, scores < 36 as severe delay of cognition.

**Table 1:** Clinical and biochemical data of study participants.

	**Control**	**HHcy**	**cblC-MMA**
Men [*n* (%)]	30(52.6)	9(60)	21(50)
Age (years, median, 95% CI)	10.0(6.7–12.0)	16.0(14.5–17.5)	6.5(3.8–9.7)^&^
Age of onset (years, median, 95% CI)	-	14.0 (12.1–15.0)	3.6 (0.8–5.5) ^&^
Family history [*n* (%)]	-	0 (0)	4 (9.5)^&^
Creatinine (μmol/L, median, 95% CI)	44 (37–48)	65.5 (57.5–128.7)^*^	31.4 (25.8–37.8) ^&^
Urea (mmol/L, median, 95% CI)	4 (3.3–4.1)	6.98 (6.62–8.73)^*^	5.06 (4.14–6.30)^*^
Proteinuria [*n* (%)]	0 (0)	12 (80)^*^	10 (23.8)^*^^&^
Hematuria [*n* (%)]	0 (0)	9(60)^*^	9 (21.4) ^*^^&^
Normal IQ or DQ [*n* (%)]	57 (100)	15 (100)	10 (23.8)^*^^&^
Mild cognitive impairment [*n* (%)]	0 (0)	0 (0)	14 (33.3)^*^^&^
Moderate cognitive impairment [*n* (%)]	0 (0)	0 (0)	12 (28.6)^*^^&^
Severe cognitive impairment [*n* (%)]	0 (0)	0 (0)	6 (14.3)^*^^&^

* p < 0.05, compared with control group;

& p < 0.05, compare with HHcy group. HHcy, non-inherited hyperhomocysteinemia. Continuous Data were shown as median with 95% CI.

Before treatment, the blood and urine samples of participants were collected. The venous blood was collected into separate gel coagulation-promoting vacuum tube (BD Vacutainer, Plymouth, UK). The specimens were centrifuged (relative centrifugal force, 2,000 g) for 10 min after quiescence clotting for about 45 min at the room temperature (22–25°C). Then the serum was put into well-sealed freezing containers and stored at −80°C within 2 h after collection. All samples were avoided repeated freeze-thaw cycles during the examination process. The samples collection and subsequent experiments were performed with the blind method.

### Serum total exosomes isolation

Serum total exosomes were isolated by exosome isolation kit (Invitrogen, Thermo Scientific, Vilnius Lithuania, cat# 4478360) following manufacturer's instructions. Briefly, 1 ml of serum sample was centrifuged (relative centrifugal force, 2,000 g) for 30 min. The supernatant was transferred into a new tube on ice. 200 μl of total exosome isolation reagent was added into the serum supernatant. The mixture was incubated at 4°C for 30 min. After incubation, the sample was centrifuged (relative centrifugal force, 10,000 g) for 10 min. The supernatant was discarded. The pellet was completely resuspended in 200 μl phosphate-buffered saline (PBS, Jiangsu KeyGen Biotech Co., Ltd, cat# KGB5001). The exosomes were stored at −80°C.

### Serum neuronal exosomes isolation

Serum neuronal exosomes were isolated with the method reported by previous studies (26, 28, 33). Briefly, 500 μl of Dulbecco's phosphate buffered saline (DPBS, Thermo Fisher Scientific, cat# 14190144) containing the inhibitor cocktails (Roche, cat# 11873580001) was added into 1 ml serum. The mixture was centrifuged (relative centrifugal force, 4,500 g) for 20 min at 4°C. The supernatant was transferred into a new tube on ice. 200 μl of total exosome isolation reagent (Invitrogen, Thermo Scientific, Vilnius Lithuania) was added into the supernatant. After mixing, the sample was incubated 1 h at 4°C. The mixture was centrifuged (relative centrifugal force, 10,000 g) for 10 min at 4°C. The supernatant was discarded. The pellet was completely resuspended in 200 μl DPBS. Each sample received 100 μl DPBS containing 3% bovine serum albumin (BSA, KeyGen Biotech, cat# KGY00810) and was incubated for 1 h at 4°C each with 2 μg of mouse anti-human NCAM antibody (Santa Cruz Biotechnology, Santa Cruz, CA, cat# SC-106), which had been biotinylated with the EZ-Link sulfo-NHS-biotin system (Thermo Scientific, cat# A39256). The mixture was put on the rotating mixer 2 h at 4°C. Then 25 μl of streptavidin-agarose resin (Thermo Scientific, cat# 20347) was added in the mixture and put on the rotating mixer 1 h at 4°C. After centrifugation at 500 g for 10 min at 4°C and removal of the supernatant, each pellet was suspended in 50 μl of 0.05 M glycine-HCl (pH 3.0) with vortex for 10 s. Then the supernatant pH was adjusted to 7.0 with 1 M Tris-HCl (pH 8.6) and was added 150 μl DPBS. The serum neuronal exosomes were stored at −80°C.

### Western blot/ confirmation of exosome collection

Sample lysate was prepared by re-suspending isolated exosomes in RIPA buffer (Beyotime, Cat#P0013B) containing protease inhibitor cocktail and loading buffer (KeyGene, Cat#KGP101X). Then sample was heated at 95°C for 10 min. The samples were resolved on 12% SDS-PAGE gels (Bio-rad) and transferred onto PVDF membranes (Pall, Cat#BSP0161). The membranes were blocked with 5% non-fat milk (Roby, Cat# RBR501-100) in TBST (Tris-buffered saline with 0.05% Tween 20) for 1.5 h and were incubated with primary antibodies in TBST overnight at 4°C. After washing the membranes 3 times for 10 min each in TBST, the membranes were incubated with secondary antibodies for 1 h. After washing, the membranes were incubated with chemiluminescent-HRP substrate (absin, Cat#abs920) according to the manufacture instructions and exposed using enhanced chemiluminescence detection system (Minichemi 610). Anti-CD63(System Biosciences, Cat# EXOAB-CD63A-1, RRID: AB_2561274), anti-TSG101(ABclonal, Cat# A1692, RRID: AB_2763744) and anti-Calnexin (abcam, Cat# ab22595, RRID: AB_2069006, NCAM (ERIC 1) antibody (Santa Cruz Biotechnology, INC, Cat# sc-106, RRID: AB_627128) were used at a dilution of 1: 1,000. Goat anti-rabbit HRP secondary antibody (Beyotime, cat# A0208, RRID: AB_2892644) was used at a dilution of 1: 20,000 in 5% non-fat milk in TBST.

### Biochemical assessment

The blood and urine samples of participants were collected. Creatinine and urea were determined using routine chemical methods by Olympus AU5821 biochemistry analyzer (Beckman coulter, Inc. USA). Homocysteine was detected by i2000 automatic immune analyzer. Propionylcarnitine (C3), free carnitine (C0) and methylmalonic acid were detected by a liquid chromatography tandem mass spectrometer (SHIMADZU LC/MS-8050, Japan).

### Determination of methylmalonic acid by tandem mass spectrometry

#### Sample preparation

50 μl of serum or exosome sample was mixed 20 μl internal standard solution (Cambridge Isotope Laboratories). The sample was put in 200 μl solution of 50:50 (by volume) methanol: acetonitrile and mixed for 2 min. The mixture was centrifuged (relative centrifugal force, 10,000 g) for 10 min. The supernatant was collected. The supernatant was evaporated to dryness in 15 min at 60°C under dry nitrogen. The residue was dissolved in 100 μl of 20:80 (by volume) methanol: deionized water.

#### LC-MS/MS procedure

Autosampler injections of 10 ul per sample were made into the LC mobile phase flow of 0.25 ml/min. Separation of MMA and MMA-d3 from the bulk of the specimen matrix was achieved by use of chromatographic column (Waters HSS T3, 1.8μm, 2.1 × 100 mm). Gradient elution of the analytes was achieved using a program with mobile phase A (methanol 0.2%formic acid) as follows: 0 to 85% B in 0.01 min, 85% B in 3 min, 85 to 10% B in 0.01 min, 10% B in 1 min, 10 to 85% B in 0.01 min, 85% B in 2 min, then back to 0% B in 0.01 min and re-equilibration for 1 min. A triple-quadrupole mass spectrometer (SHIMADZU LC/MS-8050, Japan) operated in positive ion mode (source voltage, 5500 V). The Turbo Ion Spray ionization probe operating with the turbo gas on (10 L/min, 350°C). In the selected reaction monitoring (SRM) mode, we monitored the precursor ion (m/z, 117.4) to product ion (m/z, 73.05) for MMA and precursor ion (m/z, 120.4) to product ion (m/z, 76.10) for MMA-d3, respectively. Data were acquired and processed using the software (LabSolutions LCMS Ver.5.6).

### Statistical analysis

Statistical analysis was performed using IBM SPSS software version 26.0 and GraphPad Prism5. The statistical calculations were performed with the blind method. The tests for data normality and variance homogeneity were performed before data statistical calculations. Non-parametric data were displayed as median with 95% confidence interval (CI). Non-parametric statistical analysis was used for multi-group comparisons. The relationships between methylmalonic acid in different body fluids (serum, urine, serum total exosomes and serum neuro-exosomes) or other metabolites and participants' DQ or IQ scores were performed by Kendall's tau-b correlation. To assess the performances of metabolites in diagnosis of cblC-MMA induced cognitive impairment, we computed the area under the curve (AUC), sensitivity, and specificity as well as 95% CI by the receiver operating characteristic (ROC). Then the optimal cut-off of each parameter was calculated through Youden's method. All tests were two-tailed, and the level of significance was set to *P* < 0.05.

## Results

### Baseline and biochemistry characteristics of participants

The clinical and biochemical data of participants were shown in Table 1. Our results demonstrated that the most of non-inherited HHcy patients were adolescents (onset median age: 14.0 years). There was no family history and cognitive impairment in non-inherited HHcy patients. Proteinuria and hematuria were the most common clinical manifestations of non-inherited HHcy patients. The median onset age of cblC-MMA patients was 3.6 years old. 9.5% (4/42) cblC-MMA patients had family history. 76% (32/42) cblC-MMA patients were troubled with cognitive impairments. More than 20% cblC-MMA patients suffered proteinuria and hematuria. We analyzed the biomarkers data of participants. Compared to the control group, the levels of albumin in non-inherited HHcy patients (Z = −3.107, *p* = 0.002) and cblC-MMA patients (Z = −6.826, *p* < 0.001) were markedly decreased. The levels of creatinine (Z = −2.933, *p* = 0.003) and urea (Z = −2.588, *p* = 0.01) in non-inherited HHcy patients were markedly higher than control group.

### Metabolite analysis of participants

We measured metabolites of participants. The data were presented in Table 2. The levels of propionylcarnitine (C3) in HHcy group and cblC-MMA group were significantly higher than the control group. However, there were no difference between HHcy group and cblC-MMA group. The levels of free carnitine (C0) in cblC-MMA group were markedly lower than the control group (Z = −3.349, *p* = 0.001) and HHcy group (Z = −2.084, *p* = 0.037). The levels of homocysteine of HHcy patients were significantly higher than the control group (Z = −5.928, *p* < 0.001), but lower than cblC-MMA patients (Z = −3.787, *p* < 0.001). The urinary methylmalonic acid excretion kept very low levels in control group (< 0.001 mmol/mmol creatinine) and HHcy patients (< 0.001 mmol/mmol creatinine), however, methylmalonic acid excretion significantly increased in cblC-MMA patients (median level 0.74 mmol/mmol creatinine) (Zc-_M_ = −9.041, *p* < 0.001; Z_H − M_ = −5.742, *p* < 0.001). Compared to the control group and HHcy patients, the levels of methylmalonic acid in the serum of cblC-MMA patients significantly increased (Zc-_M_ = −8.475, *p* < 0.001; Z_H − M_ = −5.709, *p* < 0.001).

**Table 2:** Metabolite analysis in the participants.

	**Control**	**HHcy**	**cblC-MMA**
methylmalonic acid in urine (mmol/mmol creatinine)	0.00 (0.00–0.00)	0.00 (0.00–0.00)	0.74 (0.58–1.14)^*^^&^
HCY in serum (μmol/L)	5.44 (4.65–5.78)	89.70 (69.74–112.49) ^*^	156.68 (150.07–171.08)^*^^&^
methylmalonic acid in serum (μmol/L)	0.05 (0.04–0.08)	0.06 (0.04–0.12)	5.73 (4.36–7.20)^*^^&^
C3 (μmol/L)	3.85 (3.10–4.34)	4.82 (3.48–7.06) ^*^	7.28 (4.87–9.11)^*^
C0 (μmol/L)	27.4 (25.3–30.5)	25.3 (21.4–28.3)	17.60 (13.9–23.3)^*^^&^
C3/C0	0.14 (0.11–0.15)	0.22 (0.14–0.32) ^*^	0.36 (0.25–0.56)^*^

* p < 0.01 compared with control group;

& p < 0.05 compared with HHcy group; HHcy, non-inherited hyperhomocysteinemia; MMA, methylmalonic acidemia; HCY, homocysteine; C3, propionylcarnitine; C0, free carnitine; C3/C0, the ratio of C3 and C0. Data were shown as median with 95% CI.

### The levels of metabolites biomarkers in different cognitive states

In this study, serum total exosomes and neuronal-derived exosomes were collected. The exosomes positive markers of CD63 and TSG101 and exosomes negative markers of Calnexin were confirmed by western blot analysis. The results showed that CD63 and TSG101 were expressed and Calnexin was not expressed in the separated pellets (Supplementary Figure 1). The NCAM content was expressed in immunoprecipitated neuro-exosomes (Supplementary Figure 2). Based on these data, we confirmed that serum total exosomes and neuronal-derived exosomes were successfully collected.

**Figure 1:**
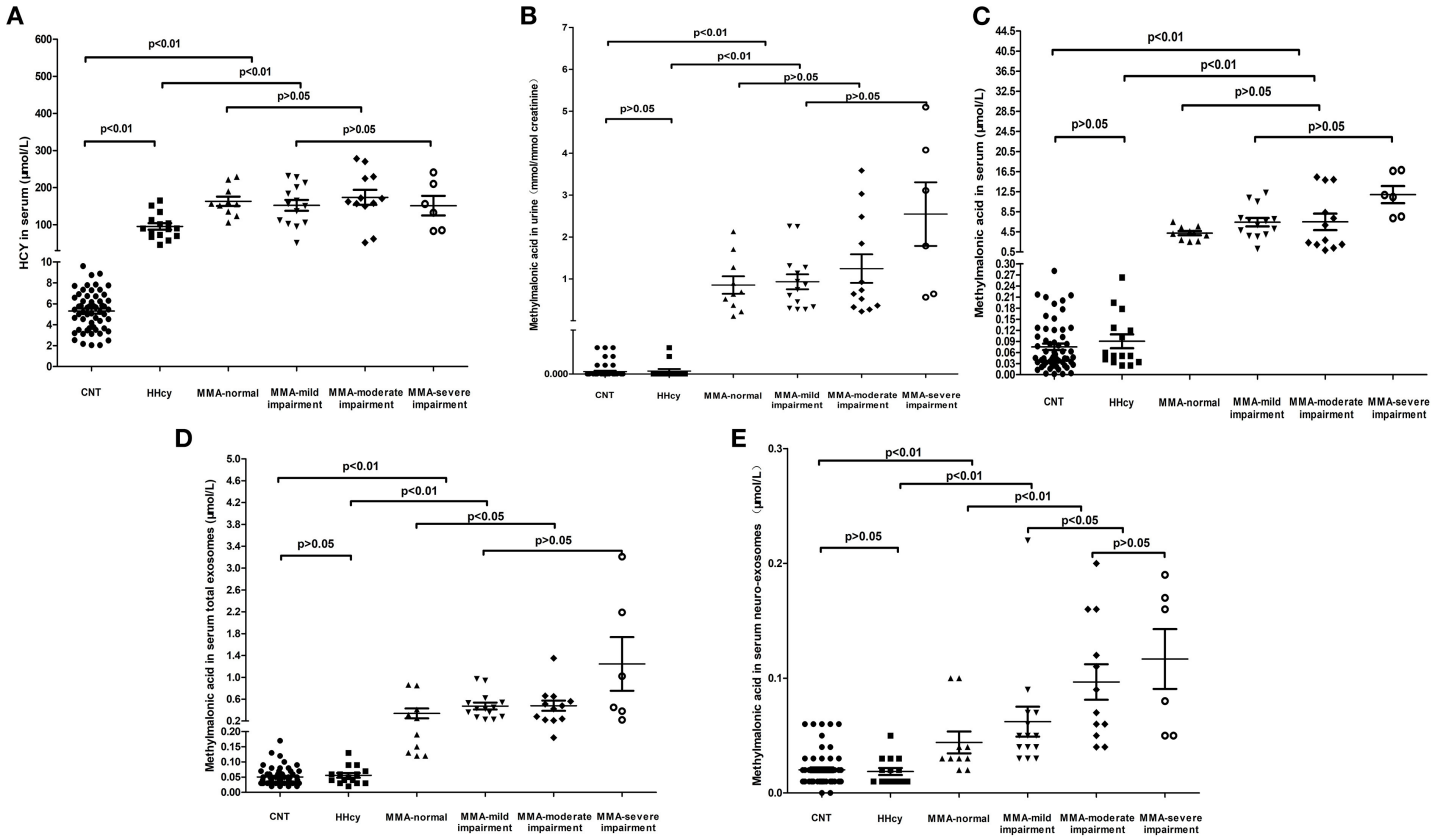
The levels of HCY in serum **(A)**, methylmalonic acid in urine **(B)**, methylmalonic acid in serum **(C)**, methylmalonic acid in serum total exosomes **(D)**, and methylmalonic acid in serum neuro-exosomes **(E)** in different groups. HCY, homocysteine; MMA, methylmalonic acidemia; CNT, Control; HHcy, non-inherited hyperhomocysteinemia; neuro-exosomes, neuronal-derived exosomes; MMA-normal, cblC type methylmalonic acidemia patients with normal IQ or DQ; MMA-mild cognitive impairment, cblC type methylmalonic acidemia patients with mild cognitive impairment; MMA-moderate cognitive impairment, cblC type methylmalonic acidemia patients with moderate cognitive impairment; MMA-severe cognitive impairment, cblC type methylmalonic acidemia patients with severe cognitive impairment.

Then, the levels of serum homocysteine, urinary methylmalonic acid, serum methylmalonic acid, methylmalonic acid in serum total exosomes and neuronal-derived exosomes in different cognitive states were analyzed. The participants included healthy children, HHcy patients and cblC-MMA patients. The healthy children and HHcy patients possessed normal cognition. According to cognitive state, cblC- MMA patients were divided into patients with cognitive impairment and patients with normal cognition. The cblC-MMA patients with cognitive impairment were further divided into three subgroups of mild, moderate and severe cognitive impairment. The levels of metabolic biomarkers of control group, HHcy group and cblC-MMA subgroups were displayed in the Figure 1. The levels of serum homocysteine (Figure 1A), urinary and serum methylmalonic acid (Figures 1B,C) in cblC-MMA patients were obviously higher than control group and HHcy group, which showed no significance between cblC-MMA patients with cognitive impairment and with normal cognition, no significance among cblC-MMA subgroups of mild, moderate and severe cognitive impairment. Then we detected the concentrations of methylmalonic acid in serum total exosomes and neuronal-derived exosomes separately. As shown in Figure 1, compare to the control group and HHcy group, the levels of methylmalonic acid in serum total exosomes (Figure 1D) and neuro-exosomes (Figure 1E) of the cblC-MMA patients markedly increased. In addition, compare to cblC-MMA patients with normal cognition, the levels of methylmalonic acid in serum total exosomes (Figure 1D) and neuro-exosomes (Figure 1E) of the cblC-MMA patients with cognitive impairment significantly increased. Especially, compared to cblC-MMA patients with mild cognitive impairment, the levels of methylmalonic acid in serum neuronal-derived exosomes (Figure 1E) markedly increased in the patients with moderate/severe cognitive impairment. But it showed no significance of the methylmalonic acid in serum total exosomes (Figure 1D)among cblC-MMA patients with different degree cognitive impairment.

### Correlation between DQ/IQ scores and the metabolites biomarkers

We analyzed the correlation between participants' DQ or IQ scores and metabolite biomarkers in the study for further exploration of the roles of these metabolites in cognitive impairment, the results were presented in Table 3. The results showed that DQ or IQ was negatively correlated with the levels of serum homocysteine, C3, methylmalonic acid in serum, urine, serum total exosomes and neuro-exosomes. The most intense association is between methylmalonic acid in urine and DQ or IQ scores (Kendall's tau-b's correlation coefficient = −0.609, *p* < 0.001). Free carnitine was positively correlated with the participants' DQ or IQ scores.

**Table 3:** Correlation between DQ or IQ scores and the metabolites biomarkers.

**Parameters**	**Kendall's tau-b's correlation coefficient**	**Kendall's tau-b's *P*-value**
HCY	−0.455	< 0.001
C3	−0.239	< 0.001
C0	0.213	0.001
C3/C0	−0.301	< 0.001
Methylmalonic acid in urine	−0.609	< 0.001
Methylmalonic acid in serum	−0.462	< 0.001
Methylmalonic acid in serum total exosomes	−0.475	< 0.001
Methylmalonic acid in serum neuro-exosomes	−0.499	< 0.001

HCY, homocysteine; C3, propionylcarnitine; C0, free carnitine; neuro-exosomes, neuronal-derived exosomes.

### The performance of metabolites biomarkers for cognitive impairment induced by cblC-MMA

To assess the capacities of biomarkers to distinguish cognitive impairment induced by cblC-MMA, ROC with AUC and 95%CI were analyzed. The performances of biomarkers for distinguishing normal children and cognitive impairment induced by cblC-MMA were showed in Table 4. In brief, the homocysteine and methylmalonic acid showed better performance than carnitines for distinguishing normal children and cblC type MMA induced-cognitive impairment.

**Table 4:** The performance of biomarkers in distinguish between normal children and cognitive impairment induced by cblC-MMA.

**Parameters**	**AUC (95%CI)**	***P*-value**
HCY	0.926 (0.875–0.976)	< 0.001
C3	0.713 (0.589–0.836)	0.001
C0	0.663 (0.535–0.791)	0.004
C3/C0	0.737 (0.617–0.856)	< 0.001
Methylmalonic acid in urine	0.941 (0.897–0.986)	< 0.001
Methylmalonic acid in serum	0.955 (0.921–0.990)	< 0.001
Methylmalonic acid in serum total exosomes	0.961 (0.924–0.999)	< 0.001
Methylmalonic acid in serum neuro-exosomes	0.921 (0.870–0.971)	< 0.001

HCY, homocysteine; C3, propionylcarnitine; C0, free carnitine; neuro-exosomes neuronal-derived exosomes.

**Figure 2:**
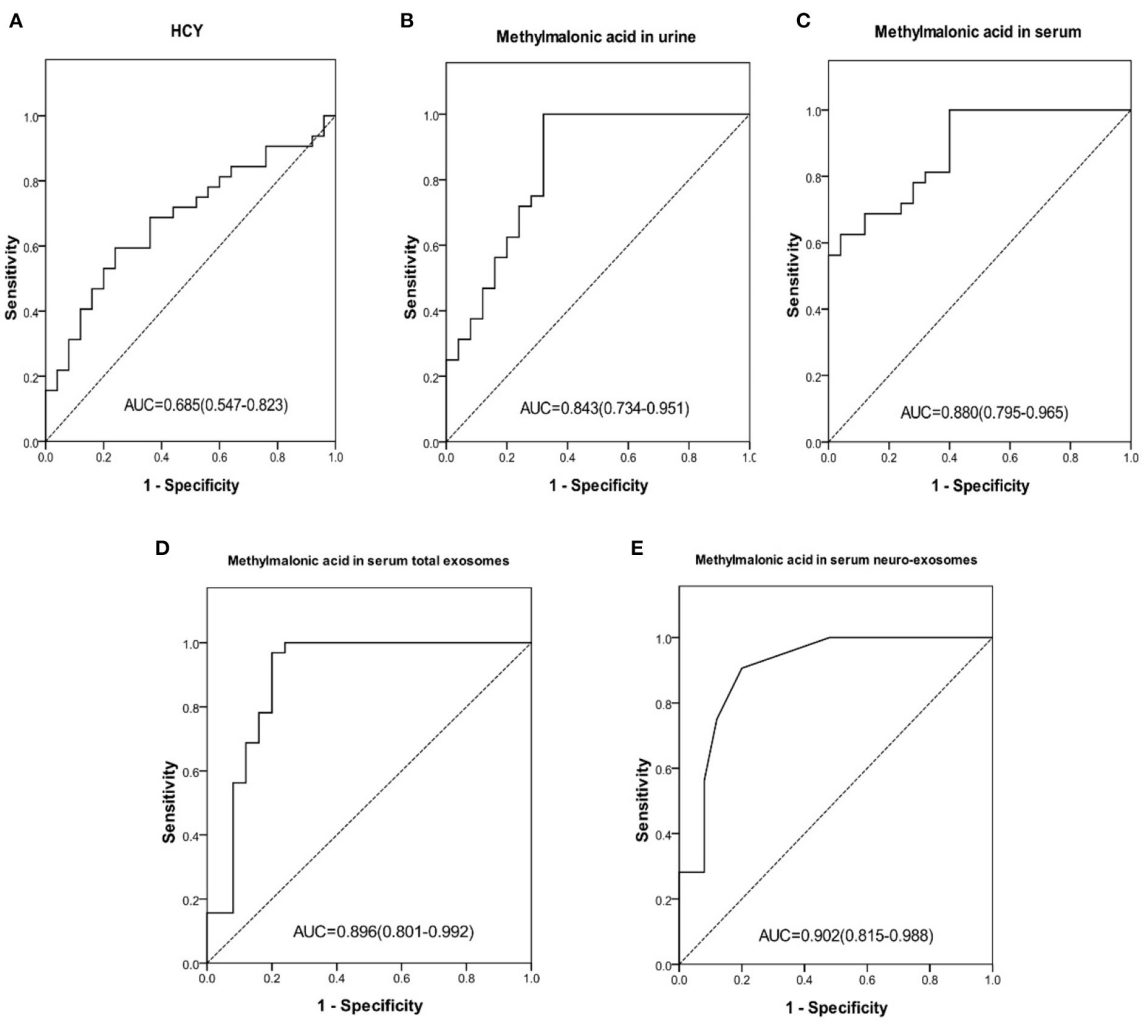
ROC curve of HCY in serum **(A)**, methylmalonic acid in urine **(B)**, methylmalonic acid in serum **(C)**, methylmalonic acid in serum total exosomes **(D)**, methylmalonic acid in serum neuro-exosomes **(E)** individually for distinguishing HHcy patients and cblC-MMA patients with cognitive impairment. ROC, receiver operating characteristic; HCY, homocysteine; neuro-exosomes, neuronal-derived exosomes; HHcy, non-inherited hyperhomocysteinemia.

To distinguish cblC-MMA patients with cognitive impairment from the HHcy patients, the performances of carnitines, homocysteine and methylmalonic acid were evaluated, the data were shown in Figure 2. Carnitine biomarkers had poor performance (AUC_c3_ = 0.541, *p* = 0.596; AUC_c0_ = 0.376, *p* = 0.111). Area under the ROC curve of serum homocysteine (Figure 2A) for discriminating cognitive impairment induced by cblC-MMA was 0.685, which decreased significantly than the diagnostic power for distinguishing cblC-MMA patients with cognitive impairment from normal children. However, the methylmalonic acid (Figures 2B–E) still had good performance.

**Figure 3:**
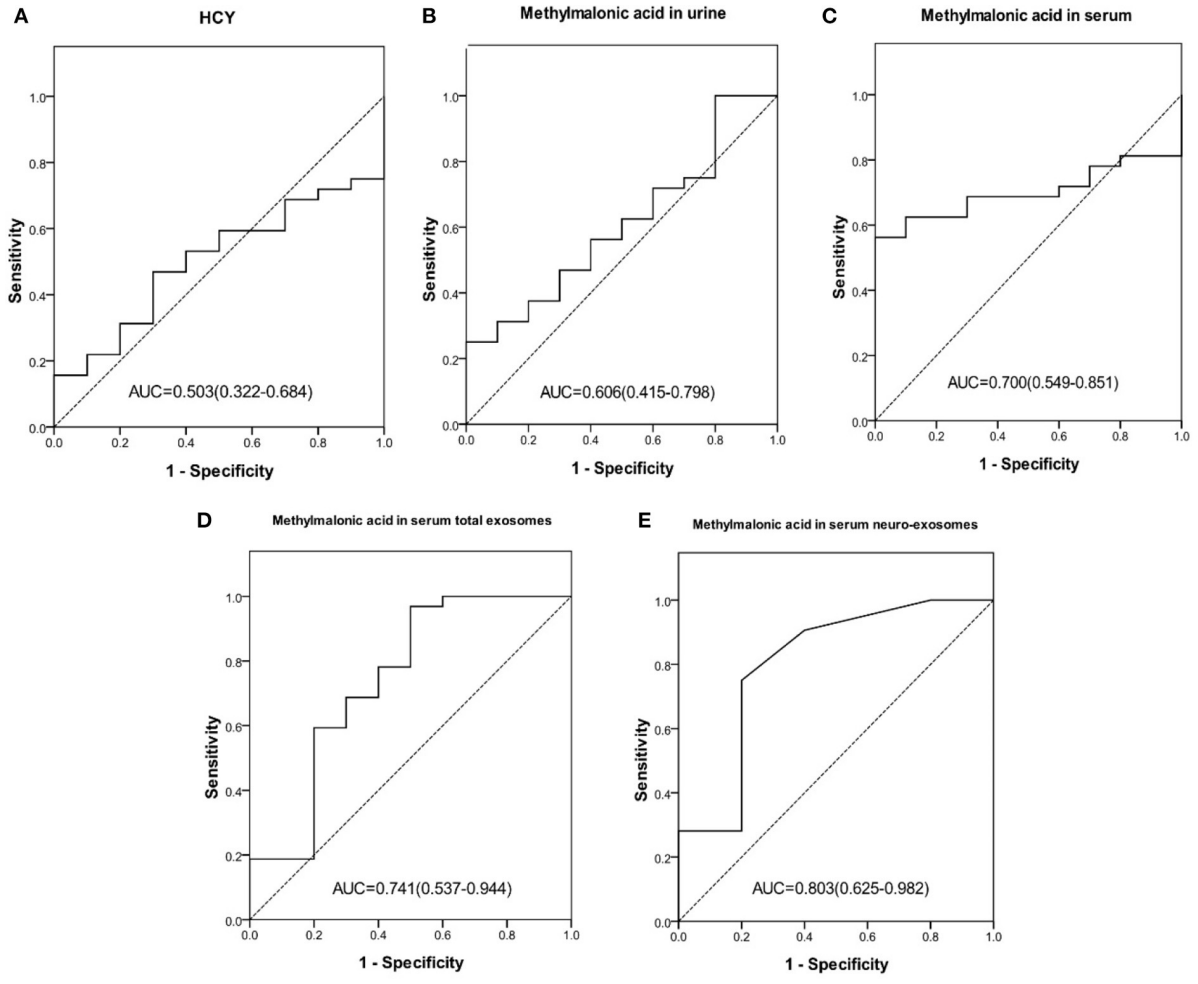
ROC curve of HCY in serum **(A)**, methylmalonic acid in urine **(B)**, methylmalonic acid in serum **(C)**, methylmalonic acid in serum total exosomes **(D)**, methylmalonic acid in serum neuro-exosomes **(E)** individually for distinguishing cblC-MMA children with cognitive impairment from without cognitive impairment. ROC, receiver operating characteristic; HCY, homocysteine; neuro-exosomes, neuronal-derived exosomes.

23.8% (10/42) cblC-MMA patients did not yet suffer cognitive impairment, other patients suffered different degrees of cognitive impairment. In order to identify children with cognitive impairment from cblC-MMA patients, the performances of biomarkers were assessed and the data were showed in Figure 3. The levels of methylmalonic acid in serum total exosomes (Figure 3D) and in serum neuro-exosomes (Figure 3E) showed better performance than serum homocysteine (Figure 3A), methylmalonic acid in the urine (Figure 3B) and in the serum (Figure 3C) for distinguishing cblC-MMA patients with or without cognitive impairment.

14 cblC-MMA patients suffered mild cognitive impairment and 18 cblC-MMA patients suffered moderate or severe cognitive impairment. Our data indicated that the levels of methylmalonic acid in serum neuro-exosomes might be helpful to discriminate cognitive impairment degrees. The AUC of methylmalonic acid in serum neuro-exosomes for distinguishing mild cognitive dysfunction from moderate or severe cognitive impairment was 0.754 (95% CI 0.578–0.930, *p* = 0.015) and the optimum cutoff value was 0.075 μmol/L, corresponding to the sensitivity of 55.6%, specificity of 85.6%, positive predictive value of 83.3% and negative predictive value of 60.0%. The levels of methylmalonic acid in urine (AUC = 0.599, *p* = 0.342), serum (AUC = 0.591, *p* = 0.382), serum total exosomes (AUC = 0.538, *p* = 0.718) had poor performance to distinguish moderate or severe cognitive impairment from mild cognitive impairment induced by cblC-MMA.

### The relationship among methylmalonic acid in different body fluids or exosomes

**Figure 4:**
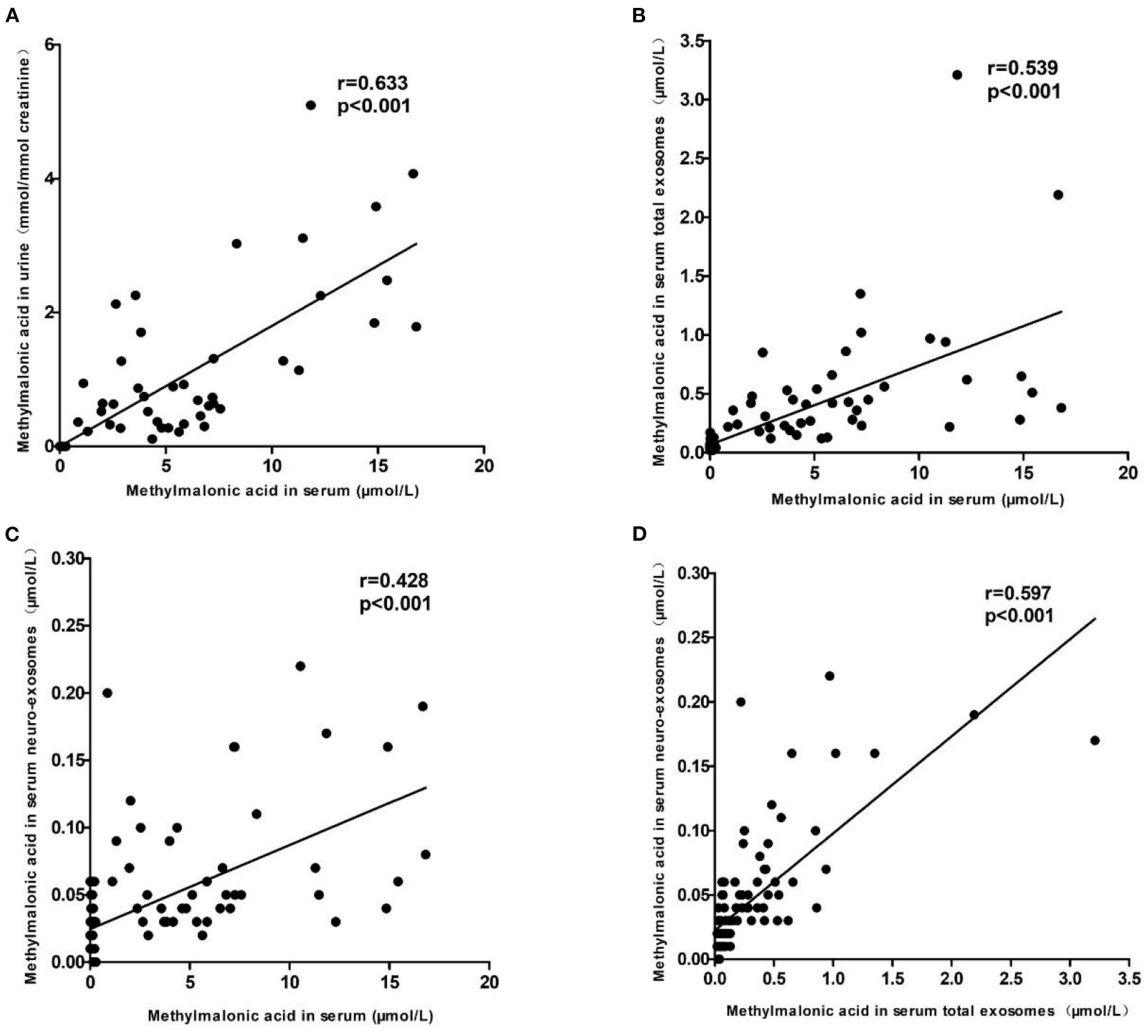
The relationship among methylmalonic acid in different body fluids or exosomes. **(A)** Showed the correlation between methylmalonic acid in urine and in serum. **(B)** Showed the correlation between methylmalonic acid in serum total exosomes and methylmalonic acid in serum. **(C)** Showed the correlation between methylmalonic acid in serum neuro-exosomes and methylmalonic acid in serum. **(D)** Showed the correlation between methylmalonic acid in serum neuro-exosomes and methylmalonic acid in serum total exosomes.

To explore the relationship among methylmalonic acid in different body fluids or exosomes, the correlation analysis was performed. The results were showed in Figure 4. The data showed that methylmalonic acid in urine is positively correlated with methylmalonic acid in serum (Figure 4A, Kendall's tau-b correlation = 0.633, *p* < 0.001). The correlations between methylmalonic acid in exosomes and methylmalonic acid in serum, respectively, were assessed using a similar model. The methylmalonic acid in serum total exosomes was associated with methylmalonic acid in serum (Figure 4B, Kendall's tau-b correlation = 0.539, *p* < 0.001) and methylmalonic acid in serum neuro-exosomes was positive correlation with methylmalonic acid in serum (Figure 4C, Kendall's tau-b correlation = 0.428, *P* < 0.001). The methylmalonic acid in serum neuro-exosomes was positive correlation with in serum total exosomes (Figure 4D, Kendall's tau-b correlation = 0.597, *p* < 0.001).

## Discussion

The cblC type MMA is the most common MMA in China. Neurological damage is the most common symptom of this disease. The biochemical metabolic characteristics of the cblC disease include the elevated serum homocysteine, the increased propionylcarnitine and the decreased free carnitine, the elevated ratio of propionylcarnitine and free carnitine and the increased methylmalonic acid levels. Hyperhomocysteinemia should be differentiated from cblC-MMA, which possesses some similar biochemical characteristics with cblC-MMA. Because the incidence of inherited hyperhomocysteinemia (34) is very low (0.02:100,000 individuals in Asians), non-inherited hyperhomocysteinemia patients were included as a comparative group in this study. In this research, 57 healthy children, 15 non-inherited HHcy patients and 42 cblC-MMA patients were recruited to explore the performances of the biomarkers for diagnosis of cognitive impairment induced by cblC-MMA.

The serum homocysteine, acylcarnitine biomarkers and methylmalonic acid had capacities to distinguish cognitive impairment induced by cblC-MMA from normal children. Then, we found non-inherited HHcy patients were not trouble with cognitive impairment, but the serum homocysteine and acylcarnitine biomarkers of non-inherited HHcy patients had similar elevated trends with cblC-MMA patients. As a result, the patients with cognitive impairment induced by cblC-MMA could not be distinguished from HHcy patients using serum homocysteine and acylcarnitine biomarkers. In addition, serum homocysteine and acylcarnitine biomarkers had a poor performance for distinguishing whether cblC-MMA patients with or without cognitive impairment or indicating the severity of cognitive impairment.

At present, methylmalonic acid is recognized as an important pathogenic substance of MMA (10). Cognitive impairment is the most common symptoms of MMA-induced brain injury. The previous study showed that 60–70% MMA patients were troubled with cognitive impairment (30, 35) and some of them had severe cognitive impairment (35).

In order to determine whether the levels of methylmalonic acid in different body fluids might be biomarkers for the diagnosis of MMA-induced cognitive impairment, the levels of methylmalonic acid in urine, serum, serum total exosomes and serum neuronal-derived exosomes were detected in healthy children, non-inherited HHcy patients and cblC-MMA patients. The results indicated that urinary methylmalonic acid had good performance to distinguish cblC-MMA patients with cognitive impairment from healthy children or non-inherited HHcy patients. However, urinary methylmalonic acid had poor performance to distinguish cblC-MMA patients with cognitive impairment from patients without cognitive impairment. As known, the levels of methylmalonic acid in urine were related to the excretion function of the kidney and the levels of serum methylmalonic acid. The kidney excretion functions of the cblC-MMA patients were affected by high concentrations of methylmalonic acid and homocysteine. The mechanisms of renal injury of cblC-MMA patients included that homocysteine caused vascular coagulation and glomerular vascular injury (36, 37) and methylmalonic acid induced renal tubular acidosis (38, 39). In this study, we found the levels of urea were obviously higher than normal children, which indicated the renal injury in cblC-MMA. Therefore, the urinary methylmalonic acid excretion was a comprehensive reflection of renal function and the levels of toxic metabolites such as serum methylmalonic acid and homocysteine, which was not a specific biomarker for the discrimination of brain injury.

In this study, the levels of the serum methylmalonic acid had good performance for distinguishing cblC-MMA induced cognitive impairment from control children or non-inherited HHcy patients. The levels of serum methylmalonic acid had poor performance to distinguish cblC-MMA patients with cognitive impairment from patients without cognitive impairment or to determine cblC-MMA patients with moderate and severe cognitive impairment, which was similar with the performance of urinary methylmalonic acid. Therefore, the levels of methylmalonic acid in serum and in urine might not accurately reflect brain injury of cblC-MMA patients. The possible reason was that methylmalonic acid in peripheral circulation could not reflect the injury of the central nervous system due to the existence of blood-brain barrier (BBB). The BBB was blood vessels that prevents the brain from exogenous and circulating toxin. The BBB played the role of a physical barrier and a metabolic barrier. The previous study reported that organic acid transporters 1 (OAT1) and 3 (OAT3) at the BBB with a low capacity for the transport of methylmalonic acid, and there was virtually no transport of methylmalonic acid across the choroid plexus (40). According to the previous study, the transport of methylmalonic acid to the brain should be a long process just relying on OAT1 and OAT3 transporter and MMA induced brain injury should be not easy to happen due to the presence of BBB. However, the brain injury was very common in MMA, which indicated that except OAT1 and OAT3 transporter, other important transport mechanism played a key role for intracerebral transport of methylmalonic acid. In addition, our previous study showed that subcutaneous continuous injection of methylmalonic acid for 24 days in normal rat cups led to the apoptosis of hippocampal neurons in rats (19), which verified that some important channels or carriers accelerated methylmalonic acid across BBB and induced brain injury.

Exosomes are vesicles that are released from cells into the extracellular space. Exosomes participate in many aspects of nervous system development and function which included regulation of nerve regeneration and synaptic communication (41). Exosomes mediate the transfer of packets of non-secreted proteins, lipids and nucleic acids within a membrane compartment. Exosomes are very important for the enclosing and transport of key proteins during developments as well as neurotoxic misfolded proteins during pathogenesis. In this study, we found the levels of methylmalonic acid in the serum total exosomes reflected the cognitive impairment induced by cblC-MMA more accurately than total methylmalonic acid in the serum. The possible reason was circulating exosomes crossed the BBB in both directions, i.e., from the bloodstream toward the brain, and from the brain to the bloodstream (42, 43), which brought more information about brain injury. In addition, the exosomes released by specific cells could package different molecules or substances from donor cells in the double membrane structure. Specially, when the special exosomes carried specific disease-related cargoes, they possessed potential uses as biomarkers. Therefore, the neuronal-derived exosomes were extracted in the serum by the established method in this study. The levels of methylmalonic acid in serum neuronal-derived exosomes were detected by LC-MS/MS, it accurately reflected existence of the cognitive impairment induced by cblC-MMA and degree of cognitive impairment severity.

In addition, if exosomes were a potential manner of methylmalonic acid transport *in vivo*, the levels of methylmalonic acid in total serum exosomes might reflect the balance of methylmalonic acid from different tissues or organs. Besides currently available cognitive assessment tests, the levels of methylmalonic acid in some special subgroup exosomes in serum derived from different tissues or organs (such as liver, kidney, heart and so on) might be helpful to diagnosis of cblC type MMA-induced injuries of different tissues and organs. It would be our further study in the future.

In summary, this study found that serum homocysteine, acylcarnitine, serum and urinary methylmalonic acid showed promise as biomarkers for distinguishing MMA-induced cognitive impairment from healthy children or non-inherited HHcy patients. More importantly, the methylmalonic acid in serum exosomes had the capacity to differentiate cblC-MMA patients with or without cognitive impairment. Especially, serum neuronal-derived exosomes reflected pathological changes in the brain of cblC-MMA patients, which should serve as a potential biomarker for estimating the severity of MMA-induced cognitive impairment.

The main limitation of the current study was the small sample size, especially we lacked a validation group, the enrollment of the patients was limited by the low prevalence of cblC type MMA (1:46,000 to 1:200,000 in European and American countries and 1:3,220 to 1:21,488 in China) (44–47). These findings warrant further investigation by more patients' enrollment and multiple center studies.

## Data availability statement

The raw data supporting the conclusions of this article will be made available by the authors, without undue reservation.

## Ethics statement

The studies involving human participants were reviewed and approved by the Medical Ethics Committee of Beijing Children's Hospital, Capital Medical University. Written informed consent to participate in this study was provided by the participants' legal guardian/next of kin.

## Author contributions

QL was responsible for the conception and design of the study. SS, HJ, YR, WS, and QL contributed to the data acquisition. SS, HJ, YR, and WS were involved in the analysis and interpretation of data. SS drafted the first version of the article. All authors critically revised the manuscript for important intellectual content and gave final approval of the version to be submitted.
