# Supplementary figures and images for: Methylmalonic acid levels in serum, exosomes, and urine and its association with cblC type methylmalonic acidemia-induced cognitive impairment
Source: Front Neurol. 2022 Dec 13;13:1090958. doi: 10.3389/fneur.2022.1090958 (PMC9792485; doi:10.3389/fneur.2022.1090958)

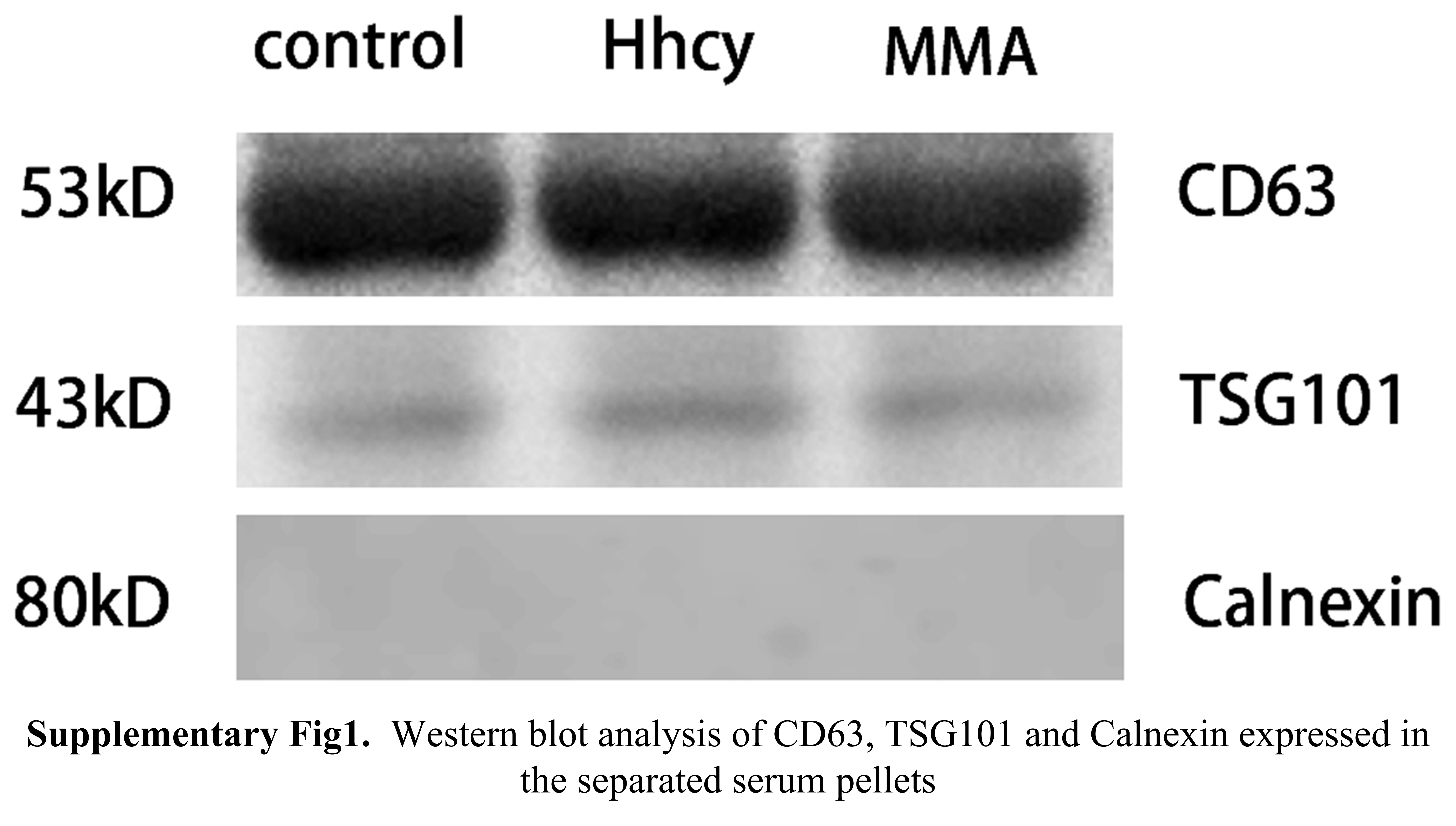

Supplement: Supplementary file 1 [file Image_1.JPEG]

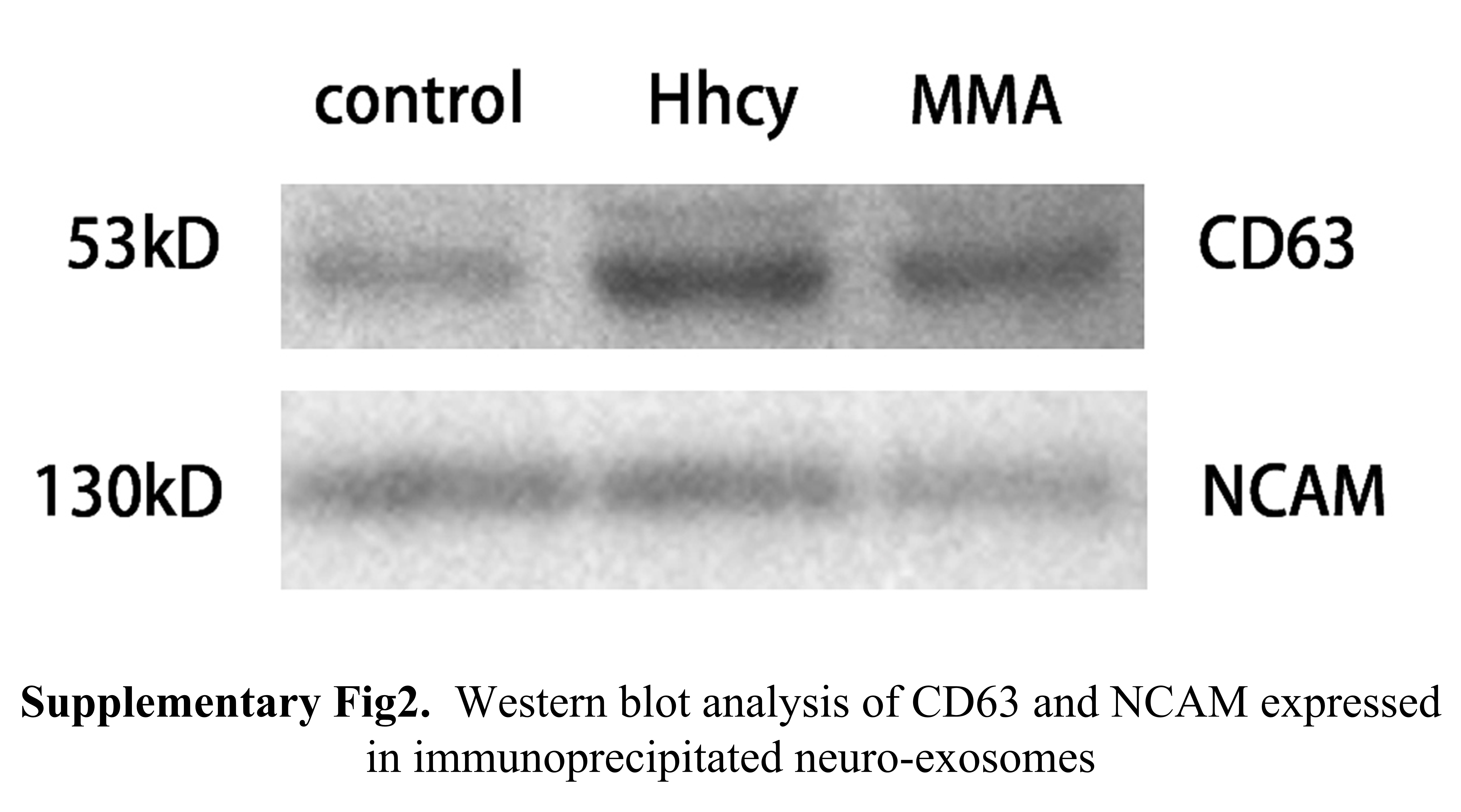

Supplement: Supplementary file 2 [file Image_2.JPEG]
